# Family history of chronic illness, preterm gestational age and smoking exposure before pregnancy increases the probability of preeclampsia in Omo district in southern Ethiopia: a case-control study

**DOI:** 10.1186/s40885-020-00149-9

**Published:** 2020-08-15

**Authors:** Kassahun Fikadu, Feleke G/Meskel, Firdawek Getahun, Nega Chufamo, Direslign Misiker

**Affiliations:** 1 Clinical Midwifery, Department of Midwifery, Arba Minch University, P.O. Box: 21, Arab Minch, Ethiopia; 2 Department of Public Health, Arbaminch University, Arab Minch, Ethiopia; 3 Department of Obstetrics and Gynecology, Arba Minch University, Arab Minch, Ethiopia

**Keywords:** Pre-eclampsia, Hospitals, Determinants, Women, Ethiopia

## Abstract

**Background:**

Preeclampsia is a complex syndrome that is considered a disorder specific to pregnancy. However, research indicates that diffuse maternal endothelial damage may persist after childbirth. On the other hand, women who had a history of pre-eclampsia are at an increased risk of vascular disease. Considering that the multifactorial nature of pre-eclampsia in a remote health setting, knowledge of risk factors of preeclampsia gives epidemiological significance specific to the study area. Therefore, this study aimed to identify the determinants of preeclampsia among pregnant women attending perinatal service in Omo district Hospitals in southern Ethiopia.

**Methods:**

An institution-based unmatched case-control study design was conducted among women visiting for perinatal service in Omo District public hospitals between February to August 2018. A total of 167 cases and 352 controls were included. Data were collected via face-to-face interviews. Bivariable and multivariable logistic regression analysis were computed to examine the effect of the independent variable on preeclampsia using Statistical Package for Social Sciences version 26 window compatible software. Variables with a *p*-value of less than 0.05 were considered statistically significant.

**Results:**

Factors that were found to have a statistically significant association with pre-eclampsia were primary relatives who had history of chronic hypertension (AOR 2.1, 95% CI: 1.06–4.21), family history of diabetes mellitus (AOR 2.35; 95% CI: 1.07–5.20), preterm gestation(AOR = 1.56, 95%CI, 1.05–2.32), and pre-conception smoking exposure (AOR = 4.16, 95%CI, 1.1–15.4).

**Conclusions:**

The study identified the risk factors for pre-eclampsia. Early detection and timely intervention to manage pre-eclampsia, and obstetric care providers need to emphasize women at preterm gestation and a history of smoking before pregnancy.

## Background

Pre-eclampsia is a unique form of life-threatening medical disorder that is clinically characterized by an elevated systolic blood pressure greater than or equal to 140 mmHg or diastolic blood pressure greater than or equal to 90 mmHg, on two occasions at least 4 h apart in a previously normotensive woman, proteinuria, or the presence of severity sign [1–3], new onset of visual disturbance, pulmonary edema, and thrombocytopenia (platelet count< 100,000/μL).

The cause of pre-eclampsia is still unknown [4]. Nevertheless, the pathogenesis of different pre-eclampsia phenotypes has not been completely elucidated [5]. Research indicates that arterial stiffness indices were found to be elevated among women who had pre-eclampsia before 34 weeks compared to normotantive mothers. This indicated that women who develop pre-eclampsia at earlier gestation exhibit impaired endothelial dysfunction [6, 7]. If detection is delayed, it progresses into a multi-organ dysfunction is more evident [8]. Recent update on preeclampsia indicates that multiple maternal organ dysfunction includes renal insufficiency, hepatic involvement, neurological or hematological complications, uteroplacental dysfunction, or fetal growth restriction may be resulted from severe preeclampsia [9].

Pre-eclampsia occurs in 3–5% of pregnancies [10]. The WHO (World Health Organization) report indicates that about 20 to 40% and 11 to 37% of pre-eclampsia occurs in women who had earlier exposure to pre-eclampsia [11]. Despite the efforts that have been made to prevent, diagnose, and manage pre-eclampsia, it continues to become a major public health problem [9, 12]. An annual report indicated that pre-eclampsia alone accounts for 70, 000 maternal deaths [13]. In women with pre-eclampsia, the risk of fetal morbidity and mortality was found to be high; indeed, it was the major cause of stillbirth and early neonatal death [14]. In Ethiopia, pre-eclampsia alone attributes 16% of pregnancy-related deaths. A report revealed an increasing trend of maternal morbidity and mortality due to pre-eclampsia [6]. Besides, it has healthcare implications for women who have preeclampsia are at increased risk of chronic illness later in life [15]. In low-income countries, especially in sub-Saharan African countries, under-reporting influences the preventive measure to lowers the burden of the disease [16].

The common factors that have been suggested to increase the risk of pre-eclampsia among women include pre-existing chronic illnesses, excessive weight gain, primiparity, advanced maternal age, first or second-degree relatives with a history of pre-eclampsia, and other environmental genetic related factors [17–19]. However, there is no single excellent factor to predict pre-eclampsia [20–22]. Although it is an inexpensive and quick way to predict the occurrence of pre-eclampsia [23, 24], in a developing nation, further investigation and validation of the existing evidence are helpful.

However, those that have been conducted have often had inadequate controls and smaller sample sizes or were not adjusted for potential confounders [18, 25, 26], this could mask the finding of previous studies. In Ethiopia, a mortality analysis conducted on mothers during the perinatal period has revealed that the new onset of pre-eclampsia has increased [6]. Therefore, this study aimed to identify the determinants of pre-eclampsia in women attending perinatal services in hospitals of Omo district, Southern Ethiopia.

## Methods

### Study area and period

A facility-based unmatched case-control study design was conducted in Omo districts in six public hospitals from February to August 2018. Based on the national 2007 census, the district houses an estimated total population of more than five and a half million [27]. The source population for this study was all women attending labor and delivery services in Arbaminch General, Konso district, Sawla district, Chencha district, Jinka General, and Gidole district hospitals. Data were collected from randomly selected pregnant women attending antenatal and delivery in all hospitals.

### Inclusion criteria

Cases were defined in two alternative ways. First, when a woman was confirmed to have an elevated blood pressure of 140/90mmHG, measured at rest or 5 min after arrival, was detected at least 4 to 6 h apart. Plus, a urine dipstick value of 2+ proteinuria and/or two random urine concentrations of 100 mg/dl collected 4 h apart after 20 completed weeks of gestation in a previously normotensive client [28], (Table 1).

**Table 1:** Diagnostic criteria used for the study on determinants of pre-eclampsia among women attending hospitals in Omo district, Southern Ethiopia, 2018

Diagnostic criteria for pre-eclampsia [28]	
The onset of symptoms after 20 weeks’ gestation with remission by 6–12 weeks postpartum^*^	
Mild pre-eclampsia:	
• Hypertension (Systolic Blood Pressure(SBP) ≥ 140 mmHg or Diastolic Blood Pressure(DBP) ≥ 90 mmHg), may be superimposed on chronic hypertension	
• Proteinuria (proteinuria ≥300 mg/24 h, or significant increase from baseline)	
Severe pre-eclampsia if one or more of the following:	
• Sustained Systolic Blood Pressure(SBP) ≥ 160 mmHg or Diastolic Blood Pressure(DPB) ≥ 110 mmHg (measured twice, at least 6 h apart)	
• Evidence of other end-organ damage	
• Deteriorating renal function including nephrotic range proteinuria ≥3 g/24 h or 3+ on urine dipstick or sudden oliguria, especially with elevated creatinine^†^	
• Central Nervous System(CNS) disturbance (altered vision, headache)	
• Pulmonary edema (3% of patients)	
• Liver dysfunction	
• Epigastric/right upper quadrant pain (stretching of hepatic capsule)	
• Thrombocytopenia (15–30% of patients)	
• HELLP syndrome is characterized by Hemolysis Elevated Liver enzymes and Low platelet count which may occur without proteinuria.	
• Evidence of fetal compromise (Intrauterine Growth Restriction-IUGR, oligohydramnios, non-reasoning fetal testing)	

Second, if the urine dipstick value became negative, the data collector also asked for the presence of severity signs. Women with elevated blood pressure and having one of the following symptoms (severe fronto-occipital headache unresponsive to antipain, right upper quadrant tenderness, epigastric pain, oliguria, upper extremity edema, facial edema, and blurring of vision) were used as an alternative to diagnose pre-eclampsia. Only if it was confirmed by a physician.

For new cases, the data collector had observed for proper measurement of blood pressure (using different parameters such as checking the functionality of the blood pressure apparatus, the tightness of the cuff comfortably over the study participant’s arm, the lower edge of the cuff was 2–3 cm above the elbow line, and whether the patient’s arm was at the level of the heart.

Controls were a woman with other cases after 20 completed weeks of gestation and who were not diagnosed with pre-eclampsia. All cases and controls were observed by the physician in the hospitals.

### Exclusion criteria

To make the study comparable, women who were not from Omo districts were excluded. In this study, women with severe medical conditions and who could not confer informed consent and with known hypertension and renal disease status were excluded from the study.

### Sample size determination

EPI INFO version 7.2 was used to compute the sample size using the double population proportion formula by assuming student occupation as a risk factor with an odds ratio of 2.65 and 5.79% among controls and exposed from the literature reviewed [29]. With corresponding assumptions of the 95% confidence interval, 5% marginal error, and 80% power, the calculated sample size was 494. Considering 5% possible non-respondents, the final sample size of 519(167 cases and 352 controls) was estimated.

### Sampling techniques and procedures

All hospitals in Omo district, such as Konso primary Hospital, Arbaminch, and Jinka General Hospitals, Chencha, Gidole, and Sawla District Hospitals, were selected purposely because of a small number of cases. For each identified hospitals, proportional to size allocation based on previous year hospital reports in a similar period of the study was considered to estimate the target population. However, due to the limited number of cases, all women who fulfilled the inclusion criteria were taken to be compared with systematically addressed controls.

**Fig. 1:**
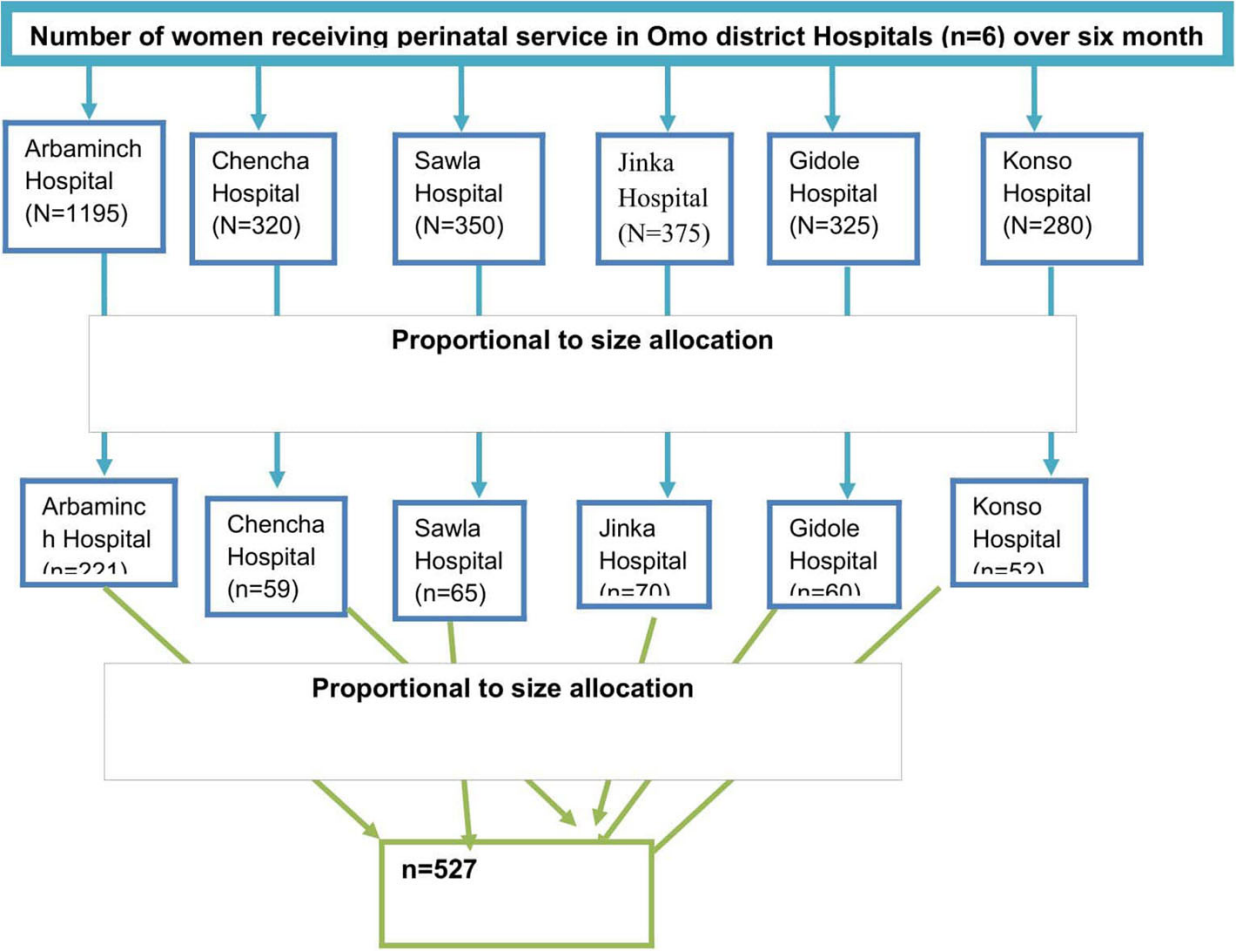
Pictorial presentation of women enrolled for a case control study on determinants of pre-eclampsia among pregnant women, Omo district hospitals,southern Ethiopia

According to the 2017 annual report, the average number of women who had attended perinatal service per month was 478, 128, 150, 130, 112, and 140 in Arbaminch General Hospital, Chencha General Hospital, Jinka Hospital, Gidole Hospital, Konso Hospital, and Sawla hospitals, respectively, (Fig. 1). Considering the average number of pregnant women attending perinatal service per day in each hospital, control were selected using a systematic random sampling technique immediately.

### Data collection procedure

Data were collected using a pre-tested, structured, and interview administered questionnaire. Besides, Patient cards were also reviewed further for the variables listed in the diagnostic criteria and to extract laboratory investigation results. The questionnaire encompasses different parts of questions related to socio-demographic variables, obstetric, medical, and behavioral-related variables. All the aforementioned variables were adopted by referring to different scholarly articles [30–38]. The questionnaires were prepared in English, translated into Amharic (Federal Government Working Language), and then translated back to English by a language expert to keep uniformity. Twelve fluent Amharic speaking Bachelor degree midwives from the health center of each town were used as data collectors. Upon arrival at the data collection site, the enumerator identified the case from antenatal and delivery booking. Then, it was cross-checked for the assurance of the diagnosis by the duty physician. The data collector also had a duty to crosscheck the diagnosis with the case definition used for this study, particularly for newly diagnosed ones.

Data were collected after the women stabilized(within 4 to 6 h of childbirth) and comfortable to respond. Six general practitioners (medical doctor) supervisors supervised the entire data collection in coordination with the principal investigator. The principal investigator coordinated the data collection team and give the necessary support. After the pre-test, the questionnaire was corrected for illogically ordered and some ambiguous and miss-leading terminologies.

### Data quality assurance

Data collectors were trained on the purpose of the study, selection of exposed and unexposed, how to keep confidentiality of patient information, the contents of the questionnaire, and data quality management by the principal investigators. The training was based on the guide that was developed for clarifying the interview administered questionnaires.

Throughout data collection, the supervisors checked the questionnaire for completeness, clarity, and consistency daily. The data collectors were oriented to correct missing data before the discharge of the patient from the hospital. Fully completed data with a few missing items were coded and duly reported to the investigator. The reliability coefficient of the tool was checked and it was 0.76. Data were entered by a trained and experienced clerk on a priory created Epi-info template.

### Data management, analysis, and interpretation

The collected data were cleaned, coded, and entered into Epi-Info version 7.2 and exported to SPSS version 26.0 for further analysis. The frequency was checked to see the accuracy, consistency, variables, and missed values. Descriptive statistics were computed and used to describe the study population using tables compared between cases and controls.

Binary and multivariable logistic regression models were fitted to identify the association between explanatory and outcome variables. Variables with a *p*-value of less than 0.20, biological plausibility, and previous study findings were considered for inclusion in the multivariable logistic regression analysis where confounders can be controlled. Multicollinearity was checked among independent variables using a variance inflation factor.

The necessary assumptions of logistic regression were made by checking Hosmer and Lemeshow goodness of fit test statistics. Variables with a *p*-value of less than 0.05 in the multivariable logistic regression analysis were considered as a statistically significant determinant. The adjusted odds ratio with the 95% CI was calculated to measure the strength of the association between the explanatory variables and the outcome variable.

## Results

### Socio-demographic characteristics of the study participants

A total of five hundred twenty-seven women were enrolled. Nine of the items had missed important variables, this gives a response rate of 98.3%. The mean age of the patients was 25 years with SD (±5.5). The majority of 110(65.9%) of cases were between 20 and 34 years of age, followed by 45(26.9%) of cases whose ages less than 20 years. The majority of 236 (67.3%) of the control groups fell in these age groups. More than 161(96%) and 234(66%) cases and controls were married. Regarding educational status, the majority of the cases (28.14%) and 30.2% of controls had completed primary education. Nearly one-fourth of cases (28.1%) and 25.3% of controls had higher education. Ninety-three 55.7% of cases were housewives compared to one hundred eighty controls, on which they had a similar role (Table 2).

**Table 2:** Sociodemographic characteristics of women attending public health hospitals in Omo District for antenatal and delivery services, Southern Ethiopia, 2018 (*n* = 519)

**Variables**	**Case** ***n*** **= 167**		**Control** ***n*** **= 352**		***P*-value**
**Age**	**No**	**%**	**No**	**%**	
Less than 20	45	26.9	80	22.7	0.19
20–34	110	65.9	237	67.3	0.39
Above 34	12	7.2	35	10	1:00
**Marital status**					
Married	161	96.4	325	92.3	0.08
Unmarried	6	3.6	27	7.7	1:00
**Residence**					
Urban	89	53.3	201	57.1	0.41
Rural	78	46.7	151	42.9	1:00
**Educational Status**					
No formal education	41	24.6	77	21.9	1:00
Primary	47	28.0	106	30.1	0.48
Secondary	39	23.4	97	27.6	0.30
College and above	40	24.0	72	20.4	0.88
**Religion**					
Orthodox	85	50.9	180	51.1	0.14
Protestant	66	39.5	142	40.3	0.10
Muslim	9	5.4	24	6.9	0.11
Others	7	4.2	6	1.7	1:00
**Maternal Occupation**					
Housewife	94	56.3	180	51.1	0.47
Merchant	26	15.6	49	13.9	0.49
Gov’t employee	15	8.9	49	13.9	0.67
Private worker	8	4.8	25	7.1	0.76
Student	16	9.6	28	8.0	0.44
Others	8	4.8	21	6.0	1:00
**Paternal Occupation**					
Gov’t employee	45	22.8	98	29.63	0.30
Merchant	43	33.5	114	28.77	0.13
Farmer	59	31.7	96	29.10	0.74
Private	8	6.6	27	6.84	0.12
Others	12	5.4	17	5.70	1:00

### Personal and family history-related characteristics

In this study, approximately 24% of patients had a family history of chronic hypertension compared to 16.8% of controls. Regarding the family history of diabetes mellitus, cases and controls had only a 3% difference, where 7.2% [11] and 10% [29] of the cases and controls had a family history of diabetes mellitus, respectively. On the other hand, the difference in the percentage of the personal history of diabetes mellitus during the last pregnancy in both cases and controls was not significant. Approximately 7 and 5% of cases and controls had diabetes mellitus during the last pregnancy, respectively. Similarly, the proportion of patients who had asthma during their last pregnancy had a 2.1 difference. Regarding the personal history of severe pre-eclampsia or eclampsia, 24(14.4%) were accounted for cases while 34(9.7%) of the controls had symptoms due to the preceding confirmed diagnosis of pre-eclampsia (Table 3).

**Table 3:** Personal and family history-related characteristics of women attending public health hospitals in Omo District for antenatal and delivery services, Southern Ethiopia, 2018 (*n* = 519)

**Variables**	**Case** ***n*** **= 167**		**Control** ***n*** **= 352**		***P*-value**
**Family history of hypertension**	**No**	**%**	**No**	**%**	
No relatives history	126	75.4	293	83.3	1:00
1st degree relatives history	19	11.4	23	6.5	0.05
2nd degree relatives history	22	13.2	36	10.2	0.23
**Family history of Diabetes Mellitus**					
Yes	12	7.2	35	9.90	0.31
No	155	92.8	317	90.1	1:00
**Personnel history of Diabetes Mellitus**					
Yes	11	6.6	19	5.4	0.59
No	156	93.4	333	94.6	1:00
**Personel history of Asthma**					
Yes	13	7.8	20	5.7	0.36
No	154	92.2	332	94.3	1:00
**Personel history of Pre-eclampsia**					
Yes	24	14.4	34	9.7	0.11
No	143	85.6	318	90.3	1:00

### Reproductive-related characteristics

More than half (50.3%) of the cases had a history of pregnancy twice comparable to 57.3% of controls. The difference in the proportion of cases and controls was equivalent to 1%. Most of the 68.9 of cases and 57.8% of the controls had preterm birth before the data collection period. The number of having had ANC follow-up at least ones during the last pregnancy was 31.7% among cases with new ANC follow-up, while it became 34.5% in controls (Table 4).

**Table 4:** Obstetric characteristics of women attending public health hospitals in Omo District for antenatal and delivery services, Southern Ethiopia, 2018 (*n* = 519)

**Variable**	**Case (*n*** **= 167)**		**Control (*n*** **= 352)**		***P*-value**
	**No**	**%**	**No**	**%**	
**Gravidity**					
Primigravida	71	42.5	127	36.2	0.12
Multigravida	84	50.3	187	57.3	0.32
Gravid multi-gravida	12	7.2	38	10.5	1:00
**Number of gestation**					
Singleton	156	93.4	326	92.9	0.74
Multiple	11	6.6	26	7.1	1:00
**Gestational Age**					
Less than 37 weeks	115	68.9	201	57.3	0.01
37 and above weeks	52	31.1	151	42.7	1:00
**ANC follow up**					
New	53	31.7	121	34.5	0.55
Repeated	114	68.3	232	65.5	1:00
**Pregnancy interval**					
No interval	63	37.7	115	32.7	0.13
Less than 2 yrs.	33	19.8	58	16.5	0.16
2 years and above	71	42.5	179	50.9	1:00
**Abortion**					
Yes	31	18.6	69	19.7	0.78
No	136	81.4	283	80.3	1:00
**Contraceptive before conception**					
No modern contraception used	88	52.7	178	50.7	1:00
Injectable	53	31.7	104	29.5	0.89
Long acting reversible	17	10.2	49	13.9	0.25
Oral pills	9	5.4	22	5.9	0.73
**Conception from a new partner**					
Yes	34	20.4	66	18.8	0.66
No	133	79.6	286	81.2	1:00
**Counseling during pregnancy**					
Yes	148	88.6	327	93.2	0.11
No	19	11.4	25	6.8	1:00
**Body Mass Index**					
Underweight	18	10.8	22	6.2	1:00
Normal	100	59.9	230	65.3	0.06
Overweight	42	25.1	94	26.8	0.10
Obese	7	4.2	6	1.7	0.58

### Behavioral and nutritional related characteristics

It was indicated that the percentage of cases who had a history of smoking before conception was 4.2%. Concerning alcohol intake during pregnancy, 48(28.7%) of cases and 89(25.4%) controls had consumed at least once. The majority, (110[65.9%] of cases and 239[68.1%] of controls had taken a cup of coffee during their course of pregnancy at least. More than 92% of both cases and controls had a habit of snack fruit while they were pregnant. Similarly, 158(94.6%) cases and 342(97.4%) controls were taken as green leafy vegetables at least once during their pregnancy. A habit to have non-strenuous physical exercise during pregnancy was responded, 83(49.7%) of cases, and 174(49.6%) controls were engaged in non-strenuous physical exercise during pregnancy.

### Determinants of preeclampsia identified

Degree of relatives with history of diabetes mellitus, gravidity, sesonality and habit of green leaf vegetable intake during antenatal follow-up were not incorporated in the multivariable analysis due to colleniearity. In model one, a family history of hypertension, women with a history of smoking before pregnancy, and a family history of diabetes were variables that found to have a significant statistical association with pre-eclampsia. However, marital status and personal stories of hypertension showed no association with pre-eclampsia. In the second model, similar variables with model one remained to have a significant statistical association with pre-eclampsia together with gestational age.

Primary relatives who had history of chronic hypertension were more than two times at increased risk for developing preeclampsia (AOR 2.1, 95% CI: 1.06–4.21).

The probability of developing preeclmpsia among women with family history of diabetes mellitus increases more than twice (AOR 2.35; 95% CI: 1.07–5.20).

Referring to gestational age at delivery, the odds of having preeclampsia increased among women who had preterm gestation(AOR = 1.56, 95%CI, 1.05–2.32) and pre-conception smoking exposure (AOR = 4.16, 95%CI, 1.1–15.4) (Table 5).

**Table 5:** Bivariable and multivariable analysis on determinants of pre-eclampsia women attending public health hospitals in Omo District for antenatal and delivery services, Southern Ethiopia, 2018 (*n* = 519)

Variable	Outcome variable		COR-95% Confidence Interval	AOR-95% Confidence Interval	*P*-value
	Pre-eclampsia	Controls			
**Family History of hypertension**					
No family history®	126(75.4%)	293(83.3%)	1:00	1:00	–
1st degree relatives history	19(11.4%)	23(6.5%)	1.92(1.01–3.65)	2.11(1.06–4.21)	0.03
2nd degree relatives history	22(13.2%)	36(10.2%)	1.42(0.80–2.51)	1.42(0.78–2.58)	0.26
**Personel History of Preeclampsia**					
Yes	24(14.4%)	34(9.7%)	1.6(0.89–2.74)	0.59(0.32–1.09)	0.09
No®	143(85.6%)	318(90.3%)	1:00	1:00	
**Family History of Diabetes Mellitus**					
Yes	12(7.2%)	35(9.90%)	0.70(0.35–1.13)	2.35(1.07–5.20)	0.03
No®	155(92.8%)	317(90.1%)	1:00	1:00	
**Marital Status**					
Married	161(96.4%)	325(92.3%)	2.23(0.90–5.51)	2.28(0.90–5.77)	0.08
Unmarried®	6(3.6%)	27(7.7%)	1:00	1:00	
**Gestational age**					
Less than 37 weeks	115(68.9%)	201(57.3%)	1.7(1.13–2.45)	1.56(1.05–2.32)	0.03
37 and Above weeks®	52(31.1%)	151(42.7%)	1:00	1:00	–
**History of Smoking before pregnancy**					
No®	96(57.5%)	216(61.4%)		1:00	–
Passive smoker	64(38.3%)	132(37.5%)	1.09(0.74–1.60)	1.14(0.77–1.67)	0.51
Active smoker	7(4.3%)	4(1.14%)	3.94(1.13–13.8)	4.16(1.12–15.4)	0.03

Note:®-reference

## Discussion

This study aimed to assess socio-demographic, medical disease, obstetric history, and behavioral determinants of pre-eclampsia. We found that having preterm gestation, and a history of smoking appeared to be a risk factor for pre-eclampsia.

A population-based study reported that preeclampsia has a strong familial tendency [39]. As a single gene hypothesis indicated, it remains an interplay of multiple factors where the effect of close relatives can be considered [40]. Besides, different scholars had tempted to characterize maternal predisposition because of genetic inheritance [41–43]. For instance, a daughter born from a pre-eclamptic mother may carry the risk for a genetic predisposition in which a gene from parents operate during uterine life through the offspring [34, 42].

Further, relatives who were not born after a pregnancy complicated by preeclampsia carries more risk [44, 45]**.** But, women born from mothers with the previous episode of preeclampsia were more likely to trigger severe preeclampsia in their pregnancy [41]. A stronger genetic predisposition may explain the clinical severity of preeclampsia [46]. Besides, strong single gene expression from the maternal side has more influence on preeclampsia than its relationship to the fetal association.

Like other studies [47, 48], this research has found a positive association with a family history of chronic hypertension and diabetes mellitus. In this study, women with a previous family history of hypertension and diabetes Mellitus had more than twice more likely to develop pre-eclampsia, respectively. Many population-based studies found a significant statistical association between pre-eclampsia and family history of chronic hypertension and diabetes mellitus. This finding is in line with studies conducted in Brazil [49], Sudan [50], Pakistan [51], Sweden [52], and Uganda [53]. Recent studies done during pregnancy noted that diabetes mellitus may involve in the development of preeclampsia in which insulin resistance may play a role in the cause of preeclampsia [54]. Several other studies revealed that women with a family history of diabetes mellitus were more likely to develop preeclampsia [38, 55–57]. It shows that family history of diabetes mellitus could be a considerable risk factor of preeclampsia.

This study showed that women at earlier gestations are at an increased risk of pre-eclampsia. Preterm gestational age attributes a higher probability of developing a severe form of pre-eclampsia than the later gestational age. This finding of this result is consistent with another study [58]. This is supported by several research findings [59, 60], in which women with PTB reported as a factor to develop PE as evidenced by many systemic inflammatory markers that appeared positive in this particular group.

Besides, an excess risk of preeclampsia was seen in women with recurrent preterm birth [58, 61]. Several other studies have suggested that early-onset and late-onset pre-eclampsia, often termed mild and severe pre-eclampsia, maybe two different entities with different causes [9, 46, 62], whereas others suggest that the risk profiles are similar for these groups [63]. In this study preterm birth was significantly higher in women with preeclampsia than without. It was indicated that 68.9% of women who had preeclampsia gave birth before 37 weeks of gestation than 57.3% of women without preeclampsia. Similar findings were observed in different parts of the world such as China [64] and Porchugese [65]. For example, a study conducted in China indicated that the difference in the rate of preeclampsia among preterm births were more than 22% compared to those without. This is supported because the risk of pre-eclampsia is inversely correlated with gestational age; the closer the gestation to 20 weeks, the more pregnant women could develop pre-eclampsia [66]. This is evidenced by a hypothesis that a common pathophysiologic mechanism, suggesting that a significant proportion of preterm births are caused by improper remodeling of the uterine spiral arteries, in earlier gestation pre-eclampsia may happen for a similar reason [67].

Another possible reason why pre-term gestation is a factor for PE may be that both conditions are due to generalized systemic inflammation, which could lead to endothelial dysfunction [68, 69]. However, it has to be underlined that neither has the possible cause and effect relationship exists between preterm gestation and preeclampsia.

Statistically speaking, our data suggest that ceasing smoking before conception did alter the risk of pre-eclampsia. Women who quit smoking before pregnancy were more than three times more likely to exhibit preeclampsia. In support of this, women who stopped smoking before pregnancy had an increased risk of pre-eclampsia compared to those who never did [70, 71]. On the other hand, women who had previous smoking exposure before pregnancy compared to non-smokers were about six times more likely to develop pre-eclampsia [72]. Research has also speculated that the probability of developing hypertension was increased among women whose ages were above 35 years [73].

The underlying mechanism that increased the risk of pre-eclampsia among women who smoked before pregnancy remains unclear. Evidence indicates that the absence of a temporary protective factor-like carbon monoxide due to low tobacco exposure could be the possible reason [72].

Evidence indicated that smoking has associated with endothelial-dependent vascular dilation [74, 75]. A decreased mRNA and protein expression of the Nitric oxide synthase activity in endothelial cells are the suggested mechanism in research from preeclamptic pregnancies [76, 77]. Women who smoke before conception may have lower nitric oxide levels [78], leads to increased vascular tension, smoking may act through this mechanism to increase the risk of preeclampsia.

Moreover, preeclamptic women have reported having more release of syncytiotrophoblastic cellular particles and syncytial cellular debris in maternal circulation, this could result from endothelial dysfunction [79–81]. In the placentas of preeclamptic women, the rate of syncytiotrophoblastic proliferation and apoptosis is higher [82]. In women with smoking exposure before conception, it has reported that an increased focal syncytial apoptosis, cytotrophoblastic hyperplasia, loss or distortion of the placental barrier, decreased syncytial pinocytotic vessels, loss of the microvilli, decreased degeneration of cytoplasmic organelles, and increased collagen in the vascular stroma are related with preeclampsia [83–86]. Evidence of syncytial damage, knots, and focal necrosis is higher in smokers [84].

Any understanding must consider the multifactorial and complex nature of pre-eclampsia pathogenesis involving genetic, environmental, and immunologic related factors. Although there is much evidence in favor of this finding, due to the smallness of cases reported smoking [26, 53], But, the association between smoking and preeclampsia should be interpreted consciously. As smoking before conception is subject to recall bias, misreporting is likely. In this regard, the true effect of smoking on preeclampsia may be masked [87, 88].

### Strengths of the study and limitations of the study

We used a clear, understandable, variety of diagnostic criteria to make the diagnosis of pre-eclampsia more accurate. We triangulate observations with a patient card review for a detailed overview of the information needed to reach the diagnosis, other than a senior physician consultation, was used. Also, we take all cases attending the hospitals included in this study during the study period.

However, we were unable to evaluate the risk of pre-eclampsia with some factors such as maternal physical activity during pregnancy, length of the sexual relationship after marriage, mechanical contraceptives before conception, and paternal smoking. Evidence indicates that there is no relationship between paternal smoking and hypertension [89].

Regardless of the findings in this study, research speculated that women who had a history of smoking with preeclampsia cause a significant adverse perinatal outcome [90]. Therefore, a pregnant woman should be advised to quit smoking. However, better awareness of the mechanism by which smoking affects the pathogenesis of preeclampsia may give insight into the upcoming therapeutic measures planned to be considered.

Considering that six hospitals in this study make the study population heterogeneous, therefore the results may not be reflective of the real condition, particularly for each setting. Besides, this study did not consider hospitals as a factor, and there may be a difference in care provision. Thus, this has to be noted for future work.

We did not consider women who might develop pre-eclampsia after 48 h to 6 weeks after delivery for two reasons. First, it was difficult to trace women after discharge because most of them had their post-partum care visits at the health center. Second, women may be delayed both from home to the health center and during referral; therefore, they may develop eclampsia fits by the time they had arrived at the hospital, which was not incorporated in this study. Due to logistic reasons, we needed to focus on pre-eclampsia alone.

## Conclusions

This study revealed that women with first degree relatives who had history of chronic hypertension, family history of diabetes mellitus, preterm gestation, and pre-conception smoking exposure were variables statistically significant determinants of pre-eclampsia in the Omo district hospitals.

Identification of women who had close relatives with history of chronic hypetenion and diabetes mellitus would be helpful in the diagnosis, monitoring, and timely mangment of women preeclampsia and its complications.

Clinicians could prompt pregnant women to avoid smoking exposure before pregnancy recognition and address barriers to perinatal care adherence during pregnancy. Besides, planning and monitoring clients are given the impact of smoking, and prematurity on pre-eclampsia could be addressed through community-based health education. Researchers should have the insight to conduct further studies on the link between smoking and pre-eclampsia.

Carefully examine preterm gestation and suspected cases of pre-eclampsia, detect and intervene as timely as possible. Working to provide enthusiastic counseling for a woman found to have a smoking history holistically may be considered during the perinatal care service provision for pregnant women. Seeking medical advice from trained health-care providers on how to cease smoking is necessary.

## Data Availability

On presumable requests, the data sets used for analysis during the current study are available from the corresponding author.

## Abbreviations

ANC: Antenatal Care
AOR: Adjusted Odds Ratio
BMI: Body Mass Index
BP: Blood Pressure
COD: Crude Odds Ratio
CI: Confidence Interval
DIC: Disseminated Intravascular Coagulation
SBP1: Systolic Blood Pressure
WHO: World Health Organization
